# A Methodological Approach to Identify Natural Compounds with Antifibrotic Activity and the Potential to Treat Pulmonary Fibrosis Using Single-Cell Sequencing and Primary Human Lung Macrophages

**DOI:** 10.3390/ijms242015104

**Published:** 2023-10-12

**Authors:** Simon H. Apte, Penny L. Groves, Maxine E. Tan, Viviana P. Lutzky, Tharushi de Silva, Joshua N. Monteith, Stephanie T. Yerkovich, Brendan J. O’Sullivan, Rohan A. Davis, Daniel C. Chambers

**Affiliations:** 1 Queensland Lung Transplant Service, The Prince Charles Hospital, Brisbane, QLD 4032, Australia; penelope.groves@health.qld.gov.au (P.L.G.); maxine.tan@health.qld.gov.au (M.E.T.); viviana.lutzky@health.qld.gov.au (V.P.L.); t.desilva@qut.edu.au (T.d.S.); brendan.osullivan2@health.qld.gov.au (B.J.O.); 2 Faculty of Medicine, The University of Queensland, Brisbane, QLD 4072, Australia; joshua.monteith@uq.net.au (J.N.M.); stephanie.yerkovich@menzies.edu.au (S.T.Y.); 3 School of Environment and Science, Griffith University, Brisbane, QLD 4111, Australia; r.davis@griffith.edu.au; 4 NatureBank, Griffith Institute for Drug Discovery, Griffith University, Brisbane, QLD 4111, Australia

**Keywords:** natural products, NatureBank, phytochemistry, rainforest plant, drug screening, lung fibrosis, alveolar macrophages, scRNA-seq, silicosis

## Abstract

Idiopathic pulmonary fibrosis (IPF) is the most common and lethal form of the interstitial pneumonias. The cause of the disease is unknown, and new therapies that stop or reverse disease progression are desperately needed. Recent advances in next-generation sequencing have led to an abundance of freely available, clinically relevant, organ-and-disease-specific, single-cell transcriptomic data, including studies from patients with IPF. We mined data from published IPF data sets and identified gene signatures delineating pro-fibrotic or antifibrotic macrophages and then used the Enrichr platform to identify compounds with the potential to drive the macrophages toward the antifibrotic transcriptotype. We then began testing these compounds in a novel in vitro phenotypic drug screening assay utilising human lung macrophages recovered from whole-lung lavage of patients with silicosis. As predicted by the Enrichr tool, glitazones potently modulated macrophage gene expression towards the antifibrotic phenotype. Next, we assayed a subset of the NatureBank pure compound library and identified the cyclobutane lignan, endiandrin A, which was isolated from the roots of the endemic Australian rainforest plant, *Endiandra anthropophagorum*, with a similar antifibrotic potential to the glitazones. These methods open new avenues of exploration to find treatments for lung fibrosis.

## 1. Introduction

Interstitial lung diseases (ILD) comprise several conditions with an estimated prevalence of ~98 per 100,000 [1] that predominantly affect the lung interstitium and share common pathological features, including interstitial scarring or fibrosis. When the cause of the ILD cannot be identified, the condition is classified as an idiopathic interstitial pneumonia, with the archetypal and most common form being idiopathic pulmonary fibrosis (IPF).

IPF is a progressive and invariably fatal disease with a global mortality rate between ~0.5 and ~12 per 100,000 and a 3- and 5-year survival rate of 61.8% and 45.6%, respectively (reviewed in [2]). The cause of IPF remains unknown; however, the consensus model centres on the notion that recurrent subclinical injury to the lung epithelium drives aberrant tissue repair mechanisms, leading to progressive fibrosis and destruction of the lung architecture. The factors that initiate and drive epithelial damage in IPF remain unknown but likely include both genetic factors and environmental exposures such as inorganic dust, allergens, air pollution, and refluxate (reviewed in [3]). Tragically, treatment options remain limited, with the only available treatment options being drugs that can, at best, slow disease progression (Pirfenidone and Nintedanib) [4]. Clearly, new therapeutics that can stop and reverse IPF progression are desperately needed.

Fibrosis is a normal step in tissue repair where fibroblasts transition to contractile myofibroblasts and drive the synthesis of extracellular matrix components, including collagen and fibronectin (reviewed in [5]). When tissue injury is transient, the fibrotic stage allows for the reorganization of the tissue architecture, after which the fibrotic lesions can be resolved. However, in the case of ongoing tissue injury in the lung due to either chronic infection, environmental insult, aberrant immune responses, genetic abnormalities such as telomeropathies [6], or microvasculature hyperpermeability (reviewed in [7,8]), fibrosis can continue to progress without resolution. Until now, it has been unclear if any of these different pathways converged at a point where common factors then continue to drive progressive and aberrant fibrosis in the lung. We are now at a point where the “omics” revolution [9] and an abundance of human lung-specific scRNA-seq data have given us clarity.

Next-generation sequencing technologies, and in particular scRNA-seq, allow for a fine-grained resolution of the phenotypes of different cell populations within human tissue. This technology has revolutionised the way we view lung macrophages, and a consensus is building in the lung fraternity that six discrete subsets exist [10,11,12,13,14], two of which are relevant here in that they appear to balance the progression of fibrosis (profibrotic macrophages) and the opposing antifibrotic population, which some have termed immunosedatory [15] or have included alveolar macrophages, which are defined by the high expression of fatty-acid-binding protein 4 (FABP4^high^) [10,14].

Macrophages are abundant in the human alveoli, comprising approximately 95% of cells in the alveolar air-space [16] and numbering around 12.5/alveolus in a healthy lung [17]. Apart from their classical role as a first line of defence in the distal lung, where they phagocytose microorganisms and other foreign particles, lung resident macrophages perform complex homeostatic roles, including immune tolerance and lung surfactant recycling. We recently showed that in humans, lung macrophages are renewed by the differentiation of blood monocyte immigrants into macrophages within the lung [18]. This, and a large body of experimental data, has suggested that lung macrophages have a degree of plasticity. 

Pulmonary fibrosis is also a common and dreaded sequalae of silicosis (reviewed in [19]). Silicosis is an ancient occupational disease caused by inhaling fine crystalline silica particles generated during mining or stone cutting and, more recently, during the fabrication of the stone benchtops that are common in most new homes. Freshly generated silica particles are toxic due to their ability to generate free radicals, and when the particles are small enough to reach the alveolus, they are rapidly phagocytosed by macrophages [20]. Whilst crystal-laden macrophages are cleared from the alveolar space, if clearance is overwhelmed, macrophages drive a chronic inflammatory state. As with all forms of pulmonary fibrosis, there are no treatment options that can stop or reverse disease progression; however, our group has initiated a program of whole-lung lavage (WLL) of silicosis patients in an effort to remove pro-inflammatory, silica-laden macrophages [21].

We have recovered, on average, 10^9^ lung macrophages per lung and have stored cells live in liquid nitrogen to make them available for research. Our preliminary unpublished results of scRNA-seq of macrophages from the lungs of silicosis patients have also found analogous profibrotic and FABP4^high^ macrophage populations consistent with the results discussed above, adding further support to the notion that although various pathways may lead to the initiation of fibrosis, once started, it is driven by aberrant profibrotic macrophages.

There remains a pressing need to find new drugs that can halt or reverse lung fibrosis; to address this need, we have developed a novel drug discovery pipeline by mining IPF scRNA-seq transcriptomic data to identify genes that delineate profibrotic and antifibrotic (FABP4^high^) macrophages with a view to identifying candidate perturbagens that could drive transcriptomic profiles away from the profibrotic phenotype and towards the antifibrotic phenotype. Then, using our unique biobank of human lung macrophages, we established an in vitro screening assay utilising cell co-culture with the test compounds followed by real-time qPCR for a set of eight signature genes. Herein, we present details of our methodological approach and provide proof-of-principle for its utility by describing assay responsiveness to drug candidates.

## 2. Results

### 2.1. Mining ScRNA-Seq Data to Identify Genes That Delineate Profibrotic and Antifibrotic (FABP4+) Macrophage Populations

Our first objective was to mine an existing scRNA-seq dataset to identify a gene set that transcriptomically characterises either profibrotic or antifibrotic macrophage subsets. We chose the dataset of Ayaub et al. [10] and Adams et al. [13] (GEO accession GSE136831 [22]). This dataset is also accessible and can be interrogated via a browser interface (ipfcellatlas.com (accessed on 25 July 2023); Rosas/Kaminski Macrophages TAB [23]).

**Figure 1:**
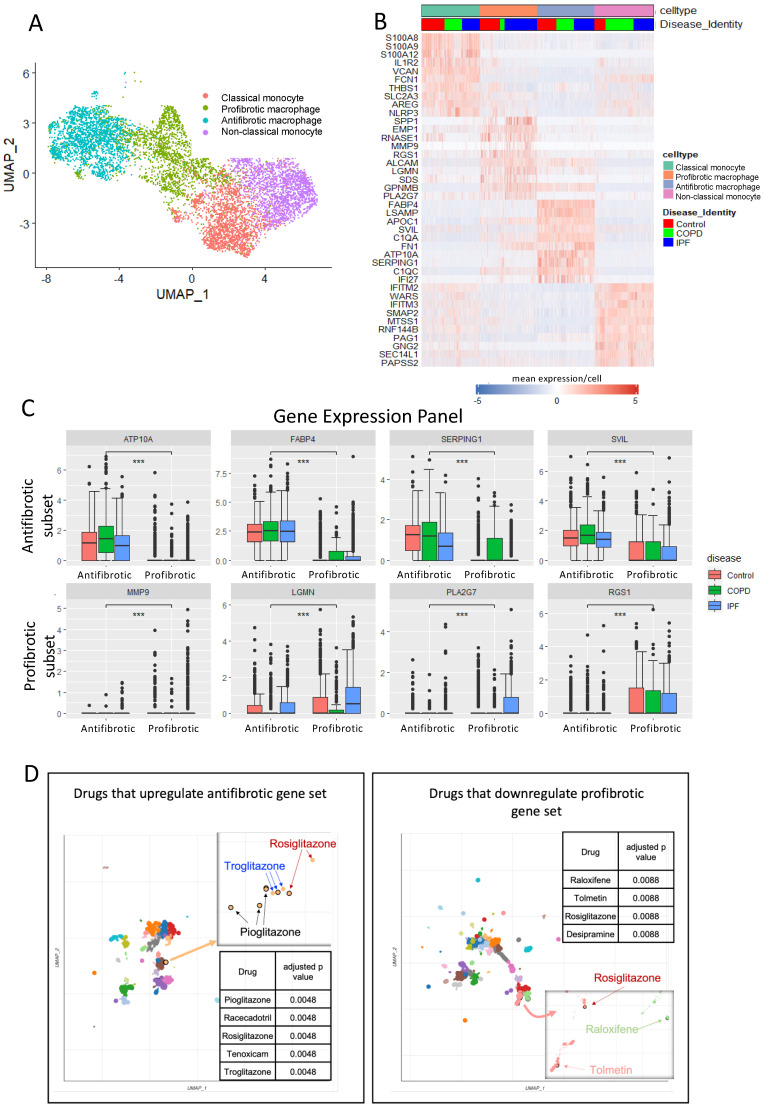
Mining scRNA-seq data to identify a gene expression panel that delineates profibrotic and antifibrotic lung macrophages and putative perturbagens. Cell clustering of the published scRNA-seq dataset (cell annotations per GEO accession GSE136831) comparing lung cells recovered from patients with IPF, COPD, and healthy controls (**A**,**B**). Gene expression panel based on the scRNA-seq dataset to delineate profibrotic and antifibrotic macrophages (**C**) (boxplots show the median and interquartile range (IQR); error bars show SE of median). Welch two-sample *t*-test comparing the control and IPF (*p*-value < 0.0005 ***). Putative perturbagens and Bokeh plots were identified using the scRNA-seq data within the Enrichr platform (**D**).

Cell clustering of macrophages and monocytes identified four distinct subsets, including classical and non-classical monocytes and antifibrotic (FABP4^hi^) macrophages and profibrotic macrophages (Figure 1A,B). After mining the dataset to find a subset of genes with significant differential expression between the profibrotic and antifibrotic macrophage subsets, we determined to use an antifibrotic panel marked by increased expression of ATP10A, FABP4, SERPING1, and SVIL and a profibrotic panel marked by increased expression of MMP9, LGMN, PLA2G7, and RGS1 (Figure 1C).

After establishing a suitable gene panel for our assay, we used the online Enrichr tool [24,25,26,27] to identify perturbagens with the potential to drive gene expression from a profibrotic profile toward an antifibrotic profile. Several perturbagens were identified with an adjusted *p*-value < 0.01 that were predicted to upregulate the antifibrotic gene set, three of which were glitazones, and they clustered together when visualised with the Enrichr cluster tool (Figure 1D—left panel); conversely, four drugs were identified that were predicted to down-regulate the profibrotic profile, of which one was also a glitazone (Figure 1D—right panel).

### 2.2. Cryopreserved Human Lung Macrophages Recovered from Whole-lung Lavage Are Viable and Amenable to In Vitro Culture

**Figure 2:**
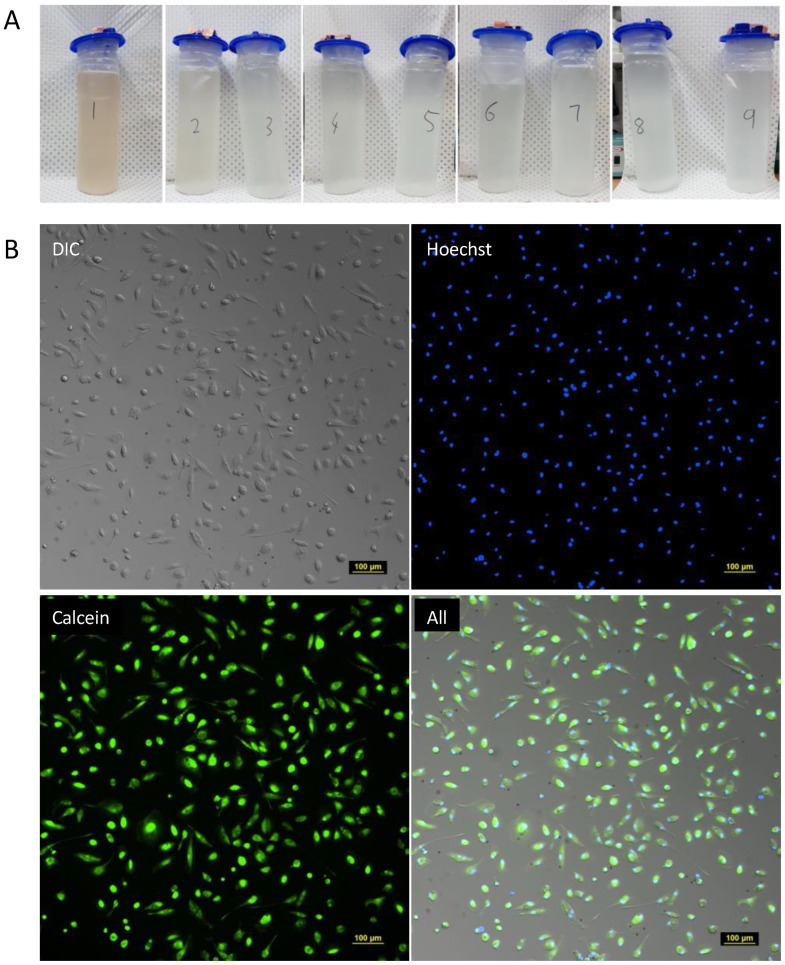
Human lung macrophages recovered from whole-lung lavage are amenable to cryopreservation and in vitro culture. Lavage fluid was collected from patients undergoing whole-lung lavage as a trial treatment for silicosis (**A**). Following processing and cryopreservation, the cells were thawed and cultured for three days prior to microscopy to assess their viability and sterility (**B**). Images were acquired with a Nikon Ti2 inverted microscope at 10× magnification; scalebar indicates 100 μm.

WLL fluid was collected from consenting patients undergoing WLL at our hospital as a trial treatment for silicosis, as reported earlier [21] (Figure 2A). Biobanked, cryopreserved lung macrophages from WLL were thawed, washed, and plated onto 96-well plates for overnight culture. The following day, non-adherent cells were gently washed away, and the media were replaced. The cells were cultured for another 3 days and then stained for fluorescent microscopy using Hoechst (nucleic acid staining) and Calcein AM (cell viability marker). Composite differential interference contrast (DIC) and fluorescence images were collected and showed the cells to be viable and without evidence of bacterial contamination within the limitations of the microscopy (Figure 2B).

### 2.3. The Human Lung Macrophage Assay Can Detect Wide-Ranging Bioactivity and Aid Discovery of Novel Antifibrotic Compounds

Next, we designed and optimised an assay to allow the most efficient cell storage and recovery, culture timing and conditions, RNA extraction and cDNA conversion, and qPCR. Our optimised method allows for the medium-throughput assessment of ~ 60 compounds in one assay round. Briefly, cryopreserved human lung macrophages are thawed and incubated overnight in 96-well plates. The following day, dead cells and media are removed by aspiration and then replaced with fresh media along with the compound of interest or controls. Following a further 3 days of incubation, media are removed by aspiration, and the cells are lysed in situ, followed by RNA extraction, cDNA synthesis, and qPCR for the eight genes detailed above and the reference gene (PPIA). Gene expression is then normalised to PPIA expression, and fold changes to the media with DMSO (no compound) control are calculated for each compound (details in Section 4).

**Figure 3:**
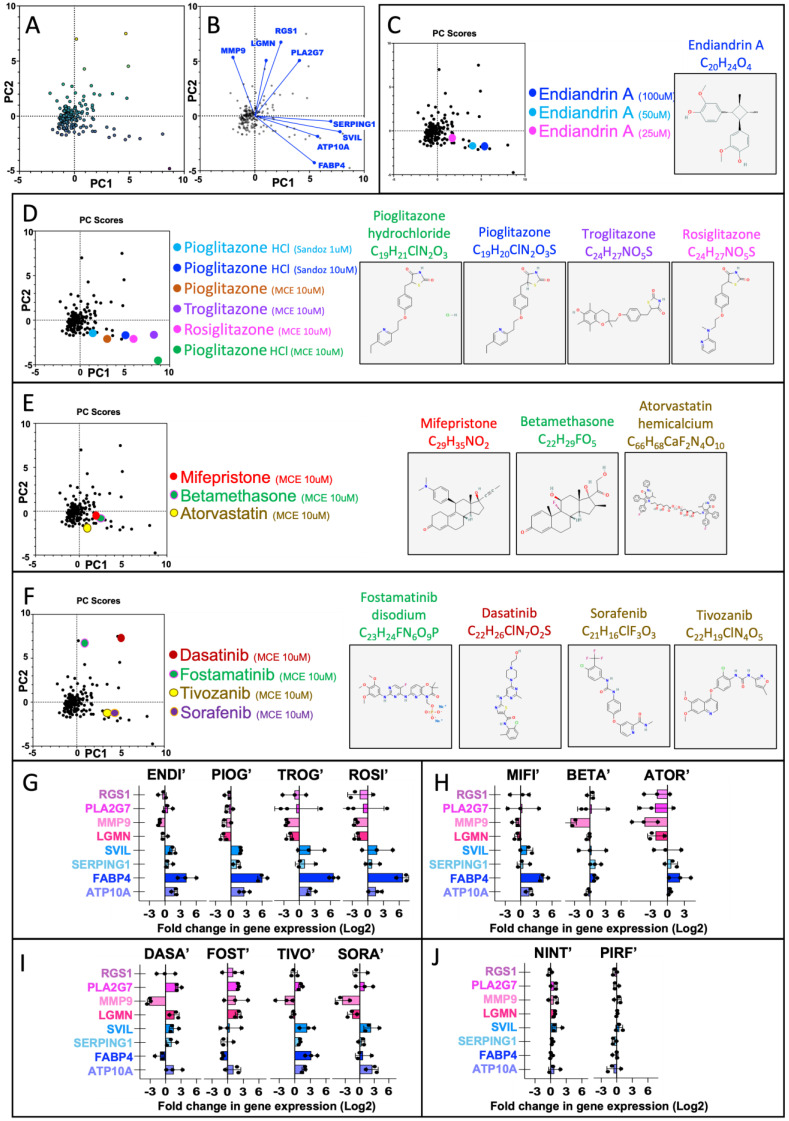
Function and utility of the human lung macrophage assay to detect the ability of putative drugs to modify the antifibrotic/profibrotic profile of lung macrophages. The fold change in gene expression of a panel of eight genes, including four that delineate an antifibrotic phenotype (ATP10A, FABP4, SERPING1, SVIL) and four that delineate a profibrotic phenotype (LGMN, MMP9, PLA2G7, RGS1), were assessed in human lung macrophages following three days in culture with test compounds (*n* = 3 different volunteers). One hundred ninety-three compounds were assayed, and PCA analysis was performed using the mean fold change in expression relative to a control sample. (**A**) PCA plot of 193 compounds and the relative PCA loadings (**B**). PCA analysis with compounds highlighted: (**C**) titration of endiandrin A; (**D**) different forms, concentrations, and preparations of glitazones; (**E**) glucocorticoid receptor agonists and one statin; (**F**) tyrosine kinase inhibitors. Two-dimensional structures from PubChem [31]. (**G**–**J**) Individual qPCR results for genes and compounds as indicated (ENDI = endiandrin A; PIOG = Pioglitazone; TROG = Troglitazone; ROSI = Rosiglitazone’ MIFI = Mifepristone; BETA = Betamethasone; ATOR = Atorvastatin; DASA = Dasatinib; FOST = Fostamatinib; TIVO = Tivozanib; SORA = Sorafenib; NINT = Nintedanib; PIRF = Pirfenidone).

Once our assay method was established, we then assayed 193 compounds (including 71 FDA-approved drugs and 122 other non-FDA compounds), including 54 pure natural products (NatureBank) and 68 compounds from the Davis Open Access Library in triplicate (*n* = 3 patients). The qPCR data for each of the eight tested genes were normalised to the reference gene PPIA using the delta CT method, and the mean of the triplicate values was assessed by principal component analysis (PCA) using GraphPad Prism software. Most compounds failed to induce large changes to gene expression and are superimposed on the PCA plot; however, 30 compounds did induce changes that can be readily observed (Figure 3A). The PCA loadings placed the antifibrotic gene set in the lower right quadrant and the profibrotic gene set in the upper two quadrants (Figure 3B). From this, we infer that compounds with the desired antifibrotic activity will appear in the lower right quadrant of the PCA.

Of the 122 non-FDA-approved compounds tested, only endiandrin A strongly modified gene expression toward the antifibrotic profile (Figure 3C,G and Table 1). The effect correlated with concentration and was toxic at 200 μM (not shown). The effect of endiandrin A was similar to that observed with glitazones, an effect predicted by the Enrichr analysis described above. Pioglitazone obtained in tablet form from our hospital pharmacy (Sandoz) and prepared by crushing and dissolving in DMSO also displayed a concentration–effect correlation and behaved similarly to Pioglitazone obtained in an FDA-approved panel (Med Chem Express). Pioglitazone HCl was the most potent compound tested, and the increased effect may be due to the higher solubility of the HCl form. The other glitazones tested (Troglitazone and Rosiglitazone) had a similar effect to Pioglitazone (Figure 3D,G and Table 1).

As endiandrin A is known to bind the glucocorticoid receptor [28], we tested two FDA-approved glucocorticoid receptor agonists (Mifepristone and Betamethasone) and both drugs had a similar activity, which was also similar to that of the lower concentration of endiandrin A (Figure 3E,H and Table 1). We were also interested if statins had any effect on gene expression, as several studies have suggested that it may delay the progression of IPF (reviewed in [29]) and it is one of the most prescribed drug classes. Atorvastatin was tested and tended to drive gene expression toward the antifibrotic profile (Figure 3E,H and Table 1).

As the tyrosine kinase inhibitor Nintedanib is one of only two drugs approved for the treatment of IPF, we tested Nintedanib and several other tyrosine kinase inhibitors, including Dasatinib, Fostamatinib, Tivozanib, and Sorafenib. Nintedanib had little effect on gene expression (not discriminated in the PCA plots but shown in Figure 3J); however, the latter two compounds had a similar effect to the glitazones, tending to drive gene expression towards an antifibrotic profile, whilst the other two drugs had the opposite effect and drove gene expression towards the profibrotic phenotype (Figure 3F,I and Table 1). These diametrically opposing results are surprising at first glance; however, it is known that different receptors and kinases can induce disparate cellular effects [30]. Finally, we tested Pirfenidone, the only other drug approved for the treatment of IPF. As with Nintedanib, Pirfenidone had very little effect on gene expression (also not able to be discriminated in the plots but shown in Figure 3J and Table 1).

## 3. Discussion

The purpose of this study was to establish a new method to identify drugs with the potential to treat IPF. At present, Pirfenidone and Nintedanib are the only drugs clinically approved for IPF. Both drugs have issues with tolerability and can, at best, only slow disease progression in some patients. New drugs are clearly required, but the factors that drive IPF are complex and likely multifactorial. The prevailing consensus model revolves around the idea that repeated injuries to the lung epithelium drive aberrant tissue repair mechanisms, leading to progressive fibrosis and destruction of lung architecture. Adding to the complexity, multiple factors have been implicated, including but not limited to dysregulated epithelial repair [3], chronic viral infection [32], cell senescence [33], aberrant fibroproliferative responses [34], and aberrant kinase activation [35]. Current drug discovery efforts focus on these areas (reviewed in [36]); however, we are proposing a different approach focussing on the re-programming of profibrotic lung macrophages to become antifibrotic. 

Recent work has redefined our understanding of human lung macrophages. Next-generation sequencing, and in particular scRNA-seq, has allowed for a fine-grained resolution of the phenotypes of lung macrophages. With this technology, it is now clear that the terminology that has been used to describe macrophages as M1 or M2 is too constricted, and the consensus is building in the IPF lung fraternity that six discrete subsets of lung macrophages exist [10,11,12,13]. Two macrophage subsets are relevant here in that they appear to balance the progression of fibrosis: a profibrotic population (IPFe or IPFexpanded [10]) and an opposing antifibrotic population that some term as alveolar macrophages and is defined by high expression of fatty-acid-binding protein 4 (FABP4) [10,11,12,13]. Furthermore, it is now apparent that the profibrotic macrophages seen in IPF also arise in patients who develop lung fibrosis following SARS-CoV-2-induced acute respiratory distress syndrome (SARS-CoV2-ARDS) [14,37,38,39]. Wendisch et al., in their study of SARS-CoV2-ARDS, went on to compare and unify the findings across different types of fibrotic lung disease [14] and firmly established that lung fibrosis is associated with a loss of antifibrotic FABP4^high^ macrophages and an expansion of profibrotic macrophages that aberrantly persist and drive fibrosis.

We have taken advantage of the burgeoning scRNA-seq transcriptomic data that are readily available for lung tissue and the consensus within multiple research groups that shows distinct subsets of lung macrophages that are either antifibrotic (FABP4^hi^) or profibrotic [10,11,12,13,14]. Using these data, we utilised the freely available tools within the Enrichr platform to predict perturbagens with the potential to drive the expression profile of lung macrophages away from the pro-fibrotic type toward the antifibrotic type. We then established a simple panel of eight gene expressions for lung macrophages, based on the scRNA-seq data, that can identify a transcriptomic shift from pro-fibrotic towards the antifibrotic phenotype.

Next, we utilised our biobank of human lung macrophages to establish and optimise a medium-throughput assay capable of testing 60 compounds in a single assay round (with *n* = 3 subjects). As predicted by the Enrichr platform, glitazones were potent modifiers of the macrophage phenotype, driving them towards the antifibrotic phenotype. This knowledge enabled us to use Pioglitazone as a positive control in all further experiments.

Once the assay was established, we began to test natural products that we had readily available. Of the 122 non-FDA-approved compounds tested, only endiandrin A (a cyclobutane lignan that binds the glucocorticoid receptor [28]) displayed a potent ability to drive macrophages toward the antifibrotic phenotype. We then began to test FDA-approved compounds utilising an FDA-Approved Drug Library Mini (96-well) as a source of compounds. 

We were able to demonstrate a high level of assay robustness; for example, different sources, preparations, and forms of Pioglitazone displayed similar characteristics, and titrated compounds displayed a linear dose–response in PCA analysis (e.g., Pioglitazone and endiandrin A in Figure 3). Furthermore, the assay displayed great utility; for example, two FDA-approved glucocorticoid receptor agonists (Mifepristone and Betamethasone) displayed a similar activity to endiandrin A, and while two tyrosine kinase inhibitors, Tivozanib and Sorafenib, were similar in activity to Pioglitazone, two others (Dasatinib and Fostamatinib) had the opposite effect and strongly shifted gene expression toward the profibrotic phenotype.

As noted above, the Enrichr analysis predicted that glitazones may drive the macrophages towards the antifibrotic phenotype. Glitazones are a class of drugs that function by activating the peroxisome proliferator-activated nuclear receptor gamma (PPARγ). They are used clinically to control type 2 diabetes by increasing insulin sensitization and increase glucose uptake. They have a wide range of other effects, including reducing pro-inflammatory cytokine production [40] and reducing hypertension and hypertriglyceridaemia (reviewed in [41,42]). PPARγ activation induces expression of FABP4 [43], so it could be predicted that glitazones would increase FABP4 in lung macrophages; however, glitazones also drove up expression of other genes delineating the antifibrotic phenotype including ATP10A, SERPING1, and SVIL, whilst lowering genes associated with the profibrotic phenotype including LGMN, MMP9, PLA2G7, and RGS1. Numerous animal studies have shown that glitazones can reduce experimentally induced hepatic fibrosis [44,45], kidney fibrosis [46], cardiac fibrosis (reviewed in [47]), and lung fibrosis [48,49]. Mechanistically, glitazones appear to inhibit profibrotic TGFβ1 signalling pathways [50] (reviewed in [42]). Furthermore, a specific antifibrotic role for FABP4 as a chaperone of nitro-fatty acids (NO_2_-FA) is emerging [51], and a recent study demonstrated reversal of bleomycin-induced lung fibrosis in a mouse model with administration of the NO_2_-FA nitro-oleic acid [52].

Our study has some limitations. First, as we are utilising a culture of biobanked, cryopreserved human lung macrophages, the macrophage phenotypes may differ from those in their native airway. Second, our assay readout is at the macro level, meaning that it is unclear how individual cells are responding to the putative drugs.

There are significant challenges to developing new drugs to treat IPF, primarily owing to the obscure origins of the disease, its unpredictable course, the diverse nature of affected patients, and the absence of reliable pharmacodynamic markers. Future strategies to develop new drugs will be aided by genomic and proteomic studies to identify genetic and protein biomarkers associated with IPF. Given the complex nature of IPF, precision medicine approaches that tailor treatments based on the specific genetic or molecular profiles of patients may improve drug efficacy. Identifying subgroups within the heterogeneous patient population may lead to more targeted and effective therapies. A further challenge is the lack of a validated animal model for IPF. It remains unclear at this point how well the widely used mouse bleomycin model of lung fibrosis reflects human IPF. Researchers use this model because it mimics several aspects of human IPF, such as inflammation, fibroblast activation, and collagen deposition in the lungs. However, like all animal models, it has limitations. While it replicates some aspects of IPF pathology, it does not fully capture the complexity of the human disease. One pressing task in this area is to align omics data from mice and humans, and we are confident this can be readily achieved for the scRNA-seq data, and in particular for the lung macrophage populations. We believe the discovery of new drugs will be expedited if we can confidently identify analogous human lung macrophage populations in the mouse.

In conclusion, herein, we designed and tested an assay based on knowledge gained from mining freely available scRNA-seq data and utilising our biobank of human lung macrophages. The assay is robust and has great utility in testing the ability of natural products and other compounds to modify lung macrophages from being profibrotic to antifibrotic. We believe the assay will be a useful tool for drug discovery and refinement.

## 4. Materials and Methods

### 4.1. ScRNA-Seq Data Mining

The Seurat v3 package (https://satijalab.org/seurat/ (accessed on 23 August 2021)) was employed for robust data analysis. Raw sequencing data underwent quality control, filtering, and normalization procedures to ensure high-quality cells. Differential gene expression analysis and the identification of cell-type-specific markers were performed using the Seurat FindMarkers function, providing insights into genes uniquely expressed in specific cell types or disease groups. Processing of the single-cell RNA-sequencing data was performed using Seurat package [53]. GEO accession GSE136831 data was processed by subletting control, IPF, and COPD myeloid cells using annotations supplied with the dataset [22]. Cells were down-sampled to 2000 cells per cell identity class, and FindallMarkers function (Wilcox method in Seurat) was used to identify differential gene expression between classes displayed as heatmap using Seurat wrapper Scillus (https://github.com/xmc811/Scillus (accessed on 23 August 2021)). Boxplots were generated using ggplot2 and ggsignif R packages and *t*-test to compare means. To select the genes to be used in the expression panel in the macrophage assay, we used the clustering shown in Figure 1 and performed differential gene expression analysis to identify cell-type-specific markers using the Seurat FindMarkers function on the GSE136831 dataset. This produced a gene list based on fold change and *p*-value. We then chose genes with the highest fold change between the antifibrotic and profibrotic subsets to derive a panel of 8 genes, including an antifibrotic panel marked by increased expression of ATP10A, FABP4, SERPING1, and SVIL and a profibrotic panel marked by increased expression of MMP9, LGMN, PLA2G7, and RGS1.

### 4.2. Collection and Cryopreservation of Human Lung Macrophages

Whole-lung lavage (WLL) fluid was collected from consenting patients undergoing WLL at our hospital as a trial treatment for silicosis, as reported earlier [21]. The lavage fluid was collected sequentially into 3-litre containers and returned to our laboratory on-site for processing. The fluid was decanted into 50 mL tubes (Sarstedt, Mawson Lakes, Australia) for centrifugation (800× *g*, 8 min) and resuspended in 20 mLs of RPMI 1640 (Gibco, Thermo Fisher Scientific, Seventeen Mile Rocks, Australia) supplemented with 2% heat-inactivated fetal calf serum (FCS) (Gibco) and penicillin/streptomycin/glutamine (Gibco), then combined with an equal volume of FCS containing 15% dimethyl sulfoxide (DMSO)(Sigma, Merck life Science, Bayswater, Australia). Cells were then cryopreserved in 40 × 1 mL aliquots in liquid nitrogen until later use.

### 4.3. Drug Discovery Assay

All cultures were performed in Dulbecco’s modified Eagle’s medium (DMEM) (Gibco) supplemented with 10% FCS (Gibco), 0.01 M Hepes solution (Sigma), and penicillin/streptomycin/glutamine (Gibco) (complete DMEM). Cryopreserved human lung macrophages from 3 patients were thawed and washed in RPMI 1640 supplemented with 2% FCS. Live cells were then counted in a haemocytometer using trypan blue stain (Gibco), resuspended in complete DMEM, and 1–1.5 × 10^5^ cells were added to 96-well flat-bottomed plates (Costar 3596, Corning, Merck, Bayswater, Australia) in 150 μL per well. Outer wells were left cell-free, and 200 μL of PBS (Gibco) was added. After incubating overnight at 37 °C in 5% CO_2_, the media were aspirated from the cells and replaced immediately with 112.5 μL complete DMEM. Compounds were diluted to 4× the desired concentration in complete DMEM, and 37.5 μL was added to specific wells individually, with the same compounds across the 3 patients’ cells (*n* = 3). Pioglitazone 1 μM was used as a positive control in subsequent assays, with media alone and media plus DMSO at the equivalent concentration of the compounds as the negative controls. After incubation for a further 3 days at 37 °C in 5% CO_2_, cells were then viewed and photographed microscopically to record the appearance of cells and viability, then all media was removed by aspiration and immediately replaced with 150 μL of RLT lysis buffer (Qiagen RNeasy96 kit). Plates were either frozen and stored at −20 °C for later RNA extraction or extracted immediately using the vacuum/centrifugation protocol supplied with the Qiagen RNeasy96 kit, with RNA eluted in 35 μL of water. All extracted RNA was then transferred to 96-well PCR plates (Applied Biosystems, Thermo Fisher Scientific, Seventeen Mile Rocks, Australia), and cDNA synthesis was performed using the QuantiTect Reverse Transcription kit (Qiagen, Clayton, Australia) in a ViiA 7 PCR machine (Applied Biosystems). The cDNA was then further diluted with at least 1× water added per sample and mixed prior to qPCR using a centrifuge vortex for PCR plates (Grant-bio). Single-plex qPCR was then performed in 384-well PCR plates (Applied Biosystems) in a Viia7 PCR machine (Applied Biosystems) using Taqman Gene Expression Master Mix (Applied Biosystems) and Taqman Gene Expression kits for PPIA housekeeping gene (Hs99999904_m1), FABP4 (Hs01086177_m1), SVIL (Hs00931004_m1), ATP10A (Hs00257114_m1), SERPING1 (Hs00163781_m1), MMP9 (Hs00957562_m1), LGMN (Hs00271599_m1), PLA2G7 (Hs00173726_m1), and RGS1 (Hs01023772_m1). Each of the test genes was then normalised to PPIA using the delta Ct method. Fold changes to the media with DMSO negative control were calculated for each compound for each of the 3 patients.

### 4.4. Compounds

All non-FDA compounds (including 54 pure natural products (NatureBank) and 68 compounds from the Davis Open Access Library) were prepared and supplied as 5 mM stock solutions in DMSO by Associate Professor Rohan Davis from NatureBank at Griffith Institute for Drug Discovery [54]. Details on the extraction and purification of endiandrin A from the roots of the rainforest plant *Endiandra anthropophagorum* have been published previously [28]. All FDA-approved compounds (apart from Pioglitazone (Sandoz, North Sydney, Australia) as noted in the text (Results Section 2.3) and below) were from the FDA-Approved Drug Library Mini (96-well) (MedChemExpress.com; Catalogue Number HY-L022) and were supplied as pre-dissolved solutions. To prepare a 10 mM stock solution of Pioglitazone HCl, a 30 mg tablet (Sandoz Australia) was crushed and dissolved in 1 mL of DMSO, heated to 37 °C for 10 min, and then vortexed, after which a clear precipitate-free solution was observed. The solution was then diluted with ultra-pure H_2_O (Invitrogen) to 3.56 mg/mL (10 mM). The Davis and NatureBank pure compound libraries were prepared by weighing out compounds into glass vials (2 mL) on a 5 decimal place balance, then adding calculated DMSO volume to each sample in order to make up a 5 mM stock solution. All DMSO stock solutions were visually inspected for precipitation or solubility issues prior to screening.

## Figures and Tables

**Table 1:** Fold changes in gene expression in lung macrophages after treatment with various compounds (Mean, (SD), *n* = 3).

Table 1	ATP10A	FABP4	SERPING1	SVIL	LGMN	MMP9	PLA2G7	RGS1
Endiandrin A	3.74 (1.27)	28.84 (32.65)	1.83 (0.98)	2.85 (1.06)	0.98 (0.42)	0.47 (0.05)	1.68 (1.02)	0.77 (0.36)
Pioglitazone	6.88 (4.66)	75.35 (47.7)	2.31 (0.93)	3.59 (0.18)	0.38 (0.15)	0.59 (0.36)	0.82 (0.45)	0.67 (0.22)
Troglitazone	5.43 (3.06)	112.25 (65.9)	3.71 (4.71)	11.02 (15.36)	0.28 (0.10)	0.18 (0.03)	6.13 (10.37)	1.04 (1.27)
Rosiglitazone	3.70 (2.69)	124.99 (70.5)	2.37 (2.25)	10.46 (14.97)	0.24 (0.07)	0.20 (0.05)	4.38 (7.40)	0.78 (1.11)
Mifepristone	3.34 (1.27)	23.11 (13.25)	1.76 (1.48)	2.74 (1.74)	0.64 (0.21)	0.53 (0.19)	4.11 (6.16)	1.47 (1.34)
Betamethasone	0.78 (0.20)	2.04 (0.49)	2.45 (1.75)	3.28 (4.66)	0.84 (0.18)	0.09 (0.06)	4.06 (6.26)	1.18 (0.45)
Atorvastatin	1.36 (0.21)	7.85 (8.57)	1.93 (1.23)	2.17 (2.92)	0.33 (0.34)	0.13 (0.16)	0.77 (1.18)	0.57 (0.70)
Dasatinib	4.47 (4.67)	0.52 (0.25)	2.63 (1.81)	3.44 (2.23)	3.72 (1.92)	0.11 (0.03)	5.94 (2.49)	1.64 (1.84)
Fostamatinib	2.81 (1.85)	0.51 (0.06)	1.11 (0.94)	2.55 (3.25)	4.29 (1.52)	6.11 (7.79)	4.10 (0.28)	3.38 (3.30)
Tivozanib	3.54 (0.86)	11.20 (8.54)	1.95 (0.24)	7.04 (5.66)	0.80 (0.17)	0.38 (0.33)	2.28 (0.62)	0.99 (0.43)
Sorafenib	7.43 (4.59)	2.42 (2.69)	1.41 (0.54)	7.47 (7.89)	0.39 (0.26)	0.11 (0.11)	3.12 (3.22)	1.43 (1.49)
Nintedanib	1.98 (1.51)	1.12 (0.22	1.27 (0.16)	2.67 (1.87)	1.73 (0.18)	1.70 (0.85)	1.71 (0.59)	0.94 (0.24)
Pirfenidone	0.87 (0.79)	0.87 (0.28)	0.73 (0.21)	1.52 (0.69)	1.04 (0.09)	1.25 (0.49)	0.98 (0.35)	0.79 (0.23)

## Data Availability

The data that support the findings of this study are available on request from the corresponding author. The data are not publicly available due to privacy or ethical restrictions.
